# Promotive Effect of Topical Ketoconazole, Minoxidil, and Minoxidil with Tretinoin on Hair Growth in Male Mice

**DOI:** 10.1155/2014/575423

**Published:** 2014-03-09

**Authors:** Muhsin A. Aldhalimi, Najah R. Hadi, Fadaa A. Ghafil

**Affiliations:** ^1^Department of Dermatology, Kufa College of Medicine, Kufa, Iraq; ^2^Department of Pharmacology and Therapeutics, Kufa College of Medicine, Kufa, Iraq

## Abstract

Recently topical use of 2% Ketoconazole solution has been reported to have a therapeutic effect on androgenic alopecia. Minoxidil is a vasodilatory medication used primarily as antihypertensive drug. It was discovered to have the side effect of hair growth and reversing baldness. Tretinoin is commonly used topically for acne treatment and in the treatment of photoaging. It is used by some as hair loss treatment. *Objective*. To compare the stimulatory effect of Ketoconazole, Minoxidil, and Minoxidil with Tretinoin on hair growth in a mouse model. *Materials and Methods*. Coat hairs on the dorsal skin of seven weeks old male mice were gently clipped and then stained by using commercial dye. These mice were divided into four groups each of five treated with topical application of ethanol 95%, Ketoconazole solution 2%, Minoxidil solution 5%, and Minoxidil with Tretinoin solution 0.1%, respectively. The drugs were applied once daily for three weeks, the clipped area was photographed, and the ratio of regrown coat area was calculated. *Results*. The results demonstrated that Ketoconazole, Minoxidil, and Minoxidil with Tretinoin had a significant stimulatory effect on hair growth compared with the control group and Minoxidil was the most effective drug among them.

## 1. Introduction

Androgenic alopecia is a partial or complete loss of hair that occurs in a progressive pattern in genetically predisposed individuals [1]. A variety of genetic and environmental factors likely play a role in androgenic alopecia, and most of contributing factors remain unknown.

The age at onset of androgenic alopecia differs but occurs usually in mid-twenties. The prevalence and severity of androgenic alopecia increase directly with age [2]. The basis of androgenic alopecia is a progressive decrease in the density of terminal hairs and concurrent increase in density of vellus hairs [3]. In effect, terminal hairs are turned off and are transformed into vellus hairs, and this effect is due to hair follicle miniaturization [4], which is associated with substantial reduction in hair diameter. Although the mechanisms of these changes have not been established definitively, male pattern baldness is known to depend on androgens, in particular the dihydrotestosterone DHT [5]. DHT is synthesized from testosterone by 5*α*-reductase enzyme type-1 and type-2, and these enzymes are found on the nuclear membranes [6].

Ketoconazole (KCZ) is an imidazole antifungal agent. Long term use of 2% KCZ was reported to be effective against androgenic alopecia in patients without seborrheic dermatitis and dandruff [7]. Minoxidil is a vasodilatory medication used primarily as antihypertensive drug. Minoxidil is a potassium channel agonist that has the chemical structure of nitric oxide (NO), a blood vessel dilator, and may be a nitric oxide agonist. This appears to explain its vasodilatory effect [8] but may also be linked to Minoxidil's ability to stimulate hair growth and treat hair loss.

Tretinoin is the acid form of vitamin A, also known as all transretinoic acid (ATRA), helps normalize hyperkeratinization, and has demonstrated significant anti-inflammatory effects. It is used by some as hair loss treatment [9].

## 2. Materials and Methods

### 2.1. Animals

The study was conducted on 20 mature male Albino-Webster mice, which were housed in the air-conditioned animal house of College of Medicine, University of Kufa, with standard animal diet and water. Their ages ranged from 6 to 7 weeks, and their weights ranged from 25 to 30 gm. The design of the study was approved by the local ethical committee in the College of Medicine, University of Kufa.

### 2.2. Methods

#### 2.2.1. Clipping of the Coat Hair of Mice

**Figure 1:**
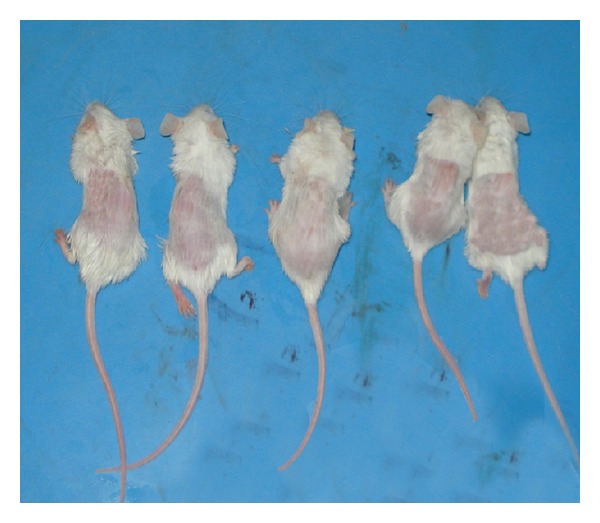
Mice after clipping of the coat hair.

One day before the experiment, the mice were anesthetized with diethyl ether, and the coat hair on the dorsal skin was gently clipped using an electric shaver to avoid injury. The skin surface of the clipped area of each mouse was observed on the day when the materials were to be applied to have a pinkish color, suggesting that it was in the resting phase [10]. Mice were randomly chosen and put into four groups, each group included five mice, and they had been photographed (Figure 1).

#### 2.2.2. Staining

**Figure 2:**
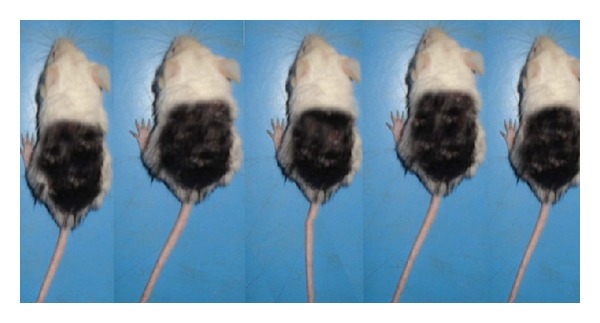
Mice after staining of the dorsal coat hair.

The denuded area of the dorsal skin of each mouse was stained by using commercial dye (Hoffmann, 2001, 2003) [11, 12]; then it was washed by using alcohol, staining done to find the ratio of area showing hair regrowth (area of white color) to the ratio of area denuded of hair (area of black color). After staining of mice, also they had been photographed (Figure 2).

#### 2.2.3. Study Groups

Group I was considered as a control group and treated with the vehicle solution (95%), ethanol alcohol, 0.1 mL applied by a micropipette to the denuded skin and then spread by means of swab once daily 6 days/weak for 3 weeks.

Group II was treated with Ketoconazole solution 2%, 0.1 mL applied to the denuded skin by a micropipette once daily for 3 weeks.

Group III was treated with Minoxidil solution 5%, 0.1 mL applied to the denuded skin by a micropipette once daily for 3 weeks.

Group IV was treated with equal amounts of Minoxidil solution 5% + Tretinoin 0.1%, 0.1 mL of each of them applied by a micropipette once daily for 3 weeks.

The mice were macroscopically observed and photographed every week until the 21st day.

*Photographic Data Analysis.* Photographic data were analyzed by a special computer program called Photoshop Visual Basic-8 program. This program calculated the ratio of the area showing hair regrowth (which was represented by the area of white color) to the ratio of the area denuded of hair (which was represented by the area of black color), and this was done for each mouse in the four groups.

### 2.3. Histological Sections

After completing the treatment for three weeks, histological sections were obtained. These sections were examined microscopically for the hair follicle number and diameter^,^ in control and treated groups.

### 2.4. Statistical Analysis

Statistical analysis was done by using one-way ANOVA test with post hoc test at level of significance *α* = 0.05 to compare between control and treated groups and then performing multiple comparisons between the treated groups.

Chi-square test had been used to compare between the proportion of histological changes in various groups.

## 3. Results

**Figure 3:**
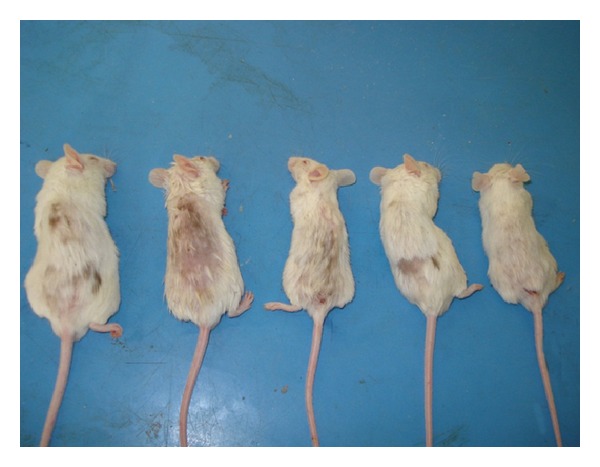
Mice treated with Ketoconazole showing significant hair growth after 3 weeks of treatment.

**Figure 4:**
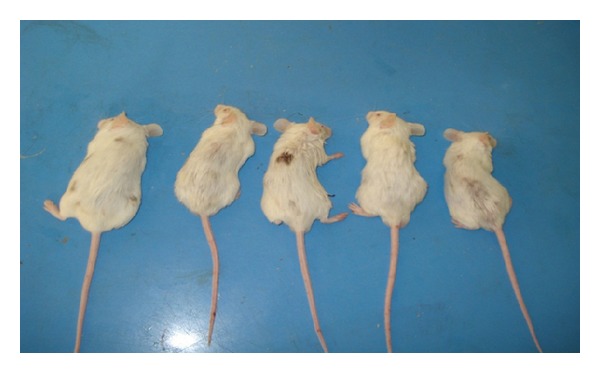
Mice treated with Minoxidil showing significant hair growth after 3 weeks of treatment.

**Figure 5:**
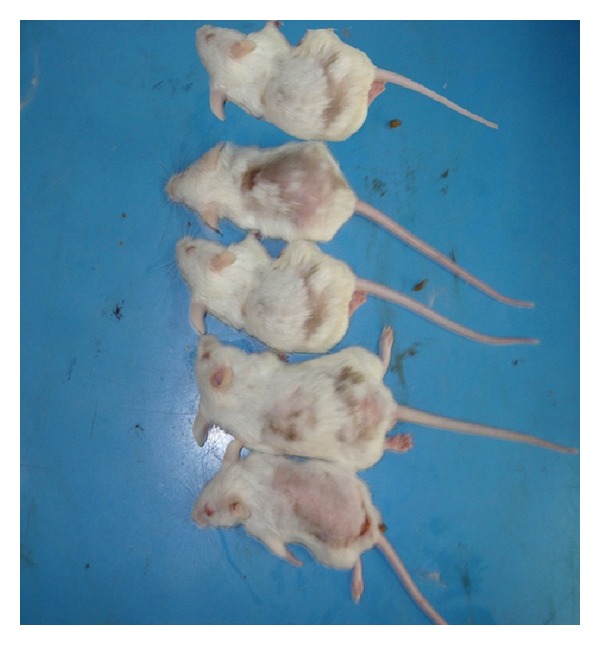
Mice treated with Minoxidil and Tretinoin showing significant hair growth after 3 weeks of treatment.

**Figure 6:**
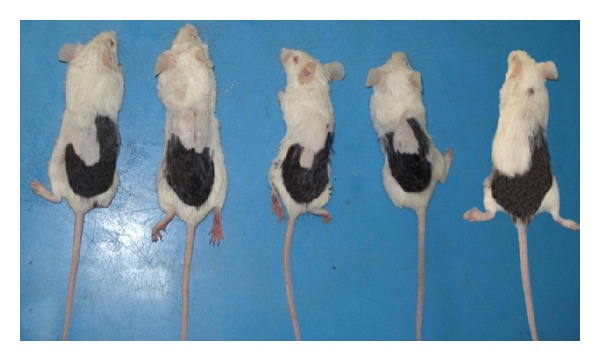
Mice of control group after 3 weeks of topical alcohol treatment showing insignificant hair growth.

There was a significant hair growth (*P* value < 0.05) in the groups treated with KCZ, Minoxidil, and Minoxidil with Tretinoin as compared with control group (Tables 1 and 2 and Figures 3, 4, 5 and 6).

**Figure 7:**
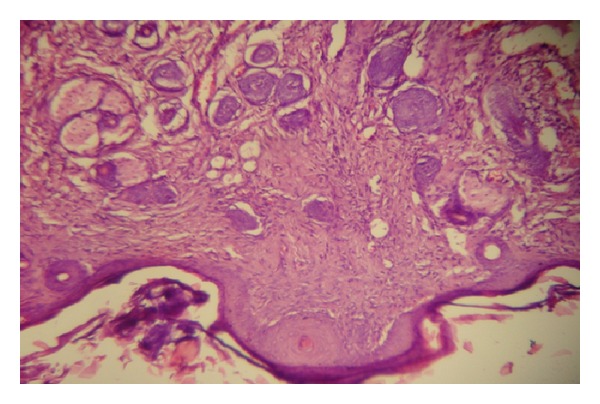
Histological section from mouse skin in control group (×40) showing normal skin layers and hair follicles.

**Figure 8:**
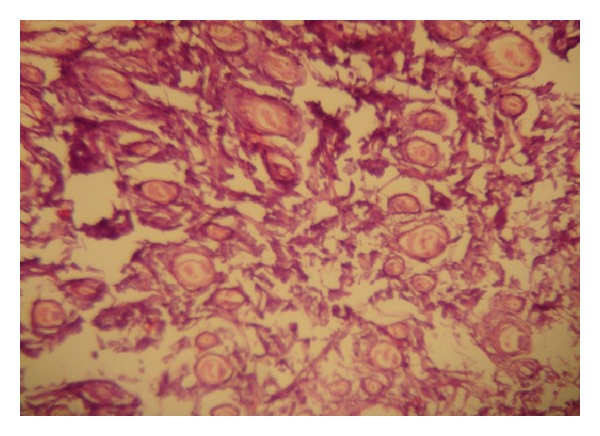
Histological section from the skin of mouse treated with Ketoconazole (×40) showing normal skin layers and increase in hair follicle diameter.

**Figure 9:**
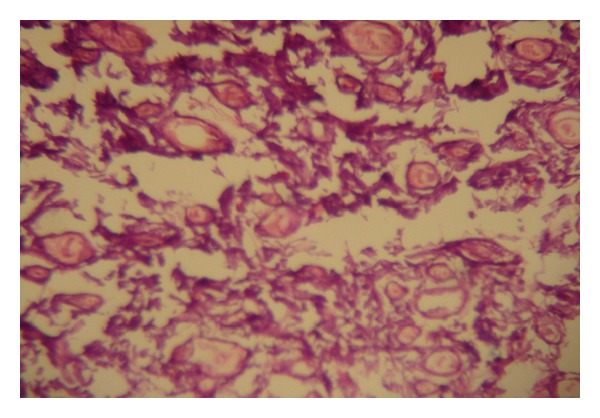
Histological section from the skin of mouse treated with Minoxidil (×40) showing normal skin layers and increase in hair follicle diameter.

**Figure 10:**
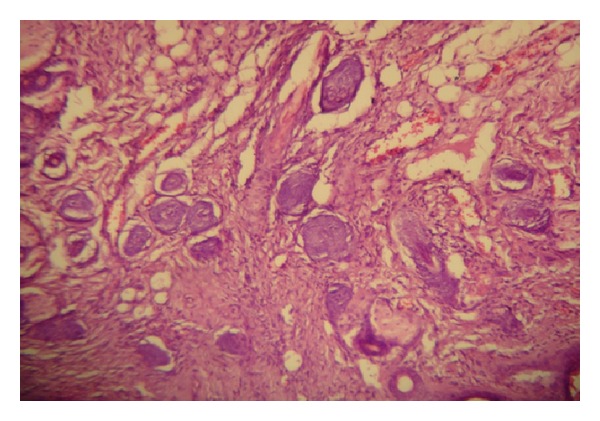
Histological section from the skin of mouse treated with Minoxidil and Tretinoin (×40) showing normal skin layers and increase in hair follicle diameter.

The histological examination of the specimens showed no significant increase in the number of hair follicles (*P* value > 0.05) in all treatment groups, while hair follicle diameter has been increased significantly (*P* value < 0.05) in all treatment groups (Tables 3, 4, and 5 and Figures 7, 8, 9, and 10).

## 4. Discussion

This study demonstrated that topical Ketoconazole stimulates hair growth significantly. This result is in agreement with Piérard-Franchimont et al. [7], Khandpur et al. [13], Jiang et al. [14], Inui and Itami [15], and Hugo Perez [16].

The efficacy of 2% KCZ shampoo in androgenic alopecia patients appeared to stem from its antiandrogenic properties [16, 17]. However, our study demonstrated that topical KCZ was effective on the androgen-insensitive coat hairs of mice, so KCZ behaved as an androgen-independent biological response modifier. However, its action on hair growth and hair follicle diameter is less than that of Minoxidil.

There was a significant increase in hair growth in the group treated with topical Minoxidil solution. This result is in agreement with Olsen et al. [18], Weiss et al. [19], and Mori and Uno [20].

Minoxidil induces rapid relaxation of vascular smooth muscle induced by its sulphated metabolite, Minoxidil sulphate [21]. The conversion of Minoxidil to Minoxidil sulphate is catalysed by sulphotransferase enzyme, which was initially demonstrated in rat liver [21] and has since been found in human liver [22], platelets [23], and mouse vibrissae follicles [24].

In scalp skin of stump-tailed macaque, sulphotransferase activity is largely localized in the hair follicle [25]. Biochemical evidence for Minoxidil sulphation by two phenol sulphotransferases has been found in human scalp skin [26].

There are individual variations in scalp sulphotransferase activity and this correlates with the level in platelets [26]. In a clinical setting scalp sulphotransferase activity was higher in men who responded to Minoxidil compared with those who did not respond [27].

In this study there was a significant hair growth in the group treated with Minoxidil and Tretinoin, and this result is in agreement with Bazzano et al. [28, 29].

Tretinoin is known to alter cell proliferation and differentiation and may promote vascular proliferation, and these actions may be important to hair growth and so affect hair follicle during the various growth and regression phases.

Preliminary studies of cRABP levels in whole scalp skin of human subjects with male pattern baldness indicated that, in the scalp areas not normally affected by alopecia, levels of cRABPs were higher than in areas with alopecia, and the levels of cRABPs in whole skin were increased by topical application of retinoic acid [30].

It has been found that combining high concentration of retinoic acid with Minoxidil causes less elongation than at low concentration suggested that retinoic acid might increase the tissue concentration of Minoxidil in hair follicles [31].

Tretinoin was reported to increase percutaneous absorption of Minoxidil by increasing the stratum corneum permeability [32]. Our study demonstrated that Tretinoin in combination with Minoxidil caused a significant increase in hair growth and a significant increase in hair follicle diameter. However, in this study the results obtained from the combination of Minoxidil with low dose Tretinoin are less than that of Minoxidil alone and this warrants further studies to evaluate the role of Tretinoin in combination with Minoxidil in the treatment of hair loss.

## Figures and Tables

**Table 1:** Mean ratio of white color (area showing hair regrowth) to the ratio of black color (area denuded of hair) after 21 days of treatment with KCZ solution 2%, Minoxidil solution 5%, and Minoxidil solution 5% with Tretinoin solution 0.1%.

Groups	Mean ratio of color difference	*P* value
Control	1.49 ± 0.17	N.S.
Ketoconazole	15.98 ± 3.42	<0.05
Minoxidil	39.53 ± 8.42	<0.05
Minoxidil and Tretinoin	15.70 ± 4.71	<0.05

All data expressed as mean ± SEM.

**Table 2:** Comparison of hair growth among the treated groups after 21 days of treatment with KCZ solution 2%, Minoxidil solution 5%, and Minoxidil solution 5% with Tretinoin solution 0.1%.

Groups	Mean difference	*P* value
KCZ-Minoxidil	−23.54 ± 7.24	<0.05
KCZ-Minoxidil and Tretinoin	0.28 ± 7.24	N.S.
Minoxidil-Minoxidil and Tretinoin	28.82 ± 7.24	<0.05

All data expressed as mean ± SEM.

**Table 3:** The mean number of hair follicles in control and treated groups examined under high power field per 10 mm after 21 days of treatment with KCZ solution 2%, Minoxidil solution 5%, and Minoxidil solution 5% with Tretinoin solution 0.1%. This table shows that hair follicle number increased insignificantly (*P* value > 0.05) in all treatment groups.

Groups	Mean number of hair follicles/10 mm	*P* value
Control	5.200 ± 1.92	
KCZ	9.600 ± 3.20	N.S.
Minoxidil	13.600 ± 2.50	N.S.
Minoxidil and Tretinoin	8.200 ± 2.58	N.S.

All data expressed as mean ± SD.

**Table 4:** The mean diameter (in micrometer) of hair follicles in control and treated groups after 21 days of treatment with KCZ solution 2%, Minoxidil solution 5%, and Minoxidil solution 5% with Tretinoin solution 0.1%.

Groups	Mean diameter of hair follicles (in *μ*m)	*P* value
Control	1.53 ± 0.09	N.S.
KCZ	2.71 ± 0.18	<0.05
Minoxidil	3.72 ± 0.20	<0.05
Minoxidil and Tretinoin	3.46 ± 0.18	<0.05

All data expressed as mean ± SEM.

**Table 5:** Comparison of the diameter (in *μ*m) of hair follicles among the treated groups after 21 days of treatment with KCZ solution 2%, Minoxidil solution 5%, and Minoxidil solution 5% with Retin—A solution 0.1%. *N* = 5 in each group.

Groups	Mean difference	*P* value
KCZ-Minoxidil	−1.01 ± 0.24	N.S.
KCZ-Minoxidil and Tretinoin	−0.75 ± 0.27	N.S.
Minoxidil-Minoxidil and Tretinoin	0.26 ± 0.28	N.S.

All data expressed as mean ± SEM.
